# Risk Factors for Gut Dysbiosis in Early Life

**DOI:** 10.3390/microorganisms9102066

**Published:** 2021-09-30

**Authors:** Kimberley Parkin, Claus T. Christophersen, Valerie Verhasselt, Matthew N. Cooper, David Martino

**Affiliations:** 1 Telethon Kids Institute, Nedlands, Perth, WA 6009, Australia; valerie.verhasselt@uwa.edu.au (V.V.); matt.cooper@telethonkids.org.au (M.N.C.); 2 School of Molecular Sciences, University of Western Australia, Nedlands, Perth, WA 6009, Australia; 3 WA Human Microbiome Collaboration Centre, School of Molecular and Life Sciences, Curtin University, Bentley, Perth, WA 6102, Australia; claus.christophersen@curtin.edu.au; 4 Centre for Integrative Metabolomics and Computational Biology, Edith Cowen University, Joondalup, Perth, WA 6027, Australia; 5 School of Medicine and Biomedical Sciences, University of Western Australia, Nedlands, Perth, WA 6009, Australia; 6 Centre for Child Health Research, University of Western Australia, Nedlands, Perth, WA 6009, Australia; 7 Murdoch Children’s Research Institute, The University of Melbourne, Parkville, VIC 3010, Australia

**Keywords:** gut microbiome, gut dysbiosis, inflammatory disease, atopy

## Abstract

Dysbiosis refers to a reduction in microbial diversity, combined with a loss of beneficial taxa, and an increase in pathogenic microorganisms. Dysbiosis of the intestinal microbiota can have a substantial effect on the nervous and immune systems, contributing to the onset of several inflammatory diseases. Epidemiological studies provided insight in how changes in the living environment have contributed to an overall loss of diversity and key taxa in the gut microbiome, coinciding with increased reports of atopy and allergic diseases. The gut microbiome begins development at birth, with major transition periods occurring around the commencement of breastfeeding, and the introduction of solid foods. As such, the development of the gut microbiome remains highly plastic and easily influenced by environmental factors until around three years of age. Developing a diverse and rich gut microbiome during this sensitive period is crucial to setting up a stable gut microbiome into adulthood and to prevent gut dysbiosis. Currently, the delivery route, antibiotic exposure, and diet are the best studied drivers of gut microbiome development, as well as risk factors of gut dysbiosis during infancy. This review focuses on recent evidence regarding key environmental factors that contribute to promoting gut dysbiosis.

## 1. Introduction

The gastrointestinal tract (GIT) is inhabited by a diverse and densely populated collection of microorganisms known as the gut microbiome, most of which are obligate anaerobes, and the majority of which belong to a small number of phyla, particularly firmicutes, bacteroidetes, and actinobacteria [1]. The gut microbiome is the most diverse and species rich of all microbiome niches in the human body. Humans and the bacteria that inhabit their GITs have coevolved to exist with a mutually beneficial symbiotic relationship. Whilst the human body acts as a well-adapted living environment, the gut microbiota provide physiological benefits to the host [2]. For example, fermentation of indigestible carbohydrates producing short chain fatty acids (SCFAs) that influence the development and function of the immune and nervous systems [3]. As well as its involvement in host health, study of the gut microbiome has been of increasing importance due to its dynamic nature; a growing body of evidence suggests it has a vital role in disease progression and prevention [4,5].

Host benefits of the gut microbiome are apparent when there is homeostasis in the relationship between the host and the gut microbiome. However, this is not always observed and a dysbiotic relationship often develops. Broadly defined, dysbiosis refers to an “imbalance” in a microbial community. This can result from a gain or loss of some of the microbial community members. This imbalance may be the result of a loss of overall diversity, a loss in beneficial microorganisms, or a gain of pathogenic species, often associated with a pathological state. Growing evidence suggests that gut dysbiosis is associated with several non-communicable diseases (for example, intestinal diseases include coeliac disease, inflammatory bowel disease, and irritable bowel syndrome, or systemic disorders such as asthma, allergy, and obesity). These diseases are increasing in prevalence in many regions of the world [6,7,8] and, as an example, thirty to forty percent of the world’s population is now affected by one or more allergic diseases [9,10], while only a few percent were affected a few decades ago. Such a high prevalence of non-communicable diseases has been linked to environmental and socioeconomic factors associated with modernisation [11]. Epidemiological studies have found a loss of overall diversity and key taxa in the gut microbiome is associated with changes in the living environment, diet and lifestyles [12]. Over the past few decades, several aspects of the human condition and environment have shifted dramatically, which has repeatedly coincided with a sustained increase in the incidence of allergies in early childhood. Specifically, factors known to influence the gut microbiome associated with modern lifestyles include sanitary improvements, increased rates of caesarean sections, smaller family sizes, sedentary lifestyles, increased usage of antibiotics, decreased *Helicobacter pylori* infections, and poorer diets [13]. While gut dysbiosis can occur at any point during life’s course, recent research has linked early gut dysbiosis with later onset of disease, highlighting the need to better understand the origins of gut dysbiosis. It remains important to understand the complex interplay between host genetics, GIT development, and environmental factors that lead to gut dysbiosis as a key preventative strategy against rising rates of non-communicable diseases. Developmental plasticity of the gut microbiome is highest in the first few years of life and evidence suggests this is a period of heightened susceptibility to dysbiosis [14].

With the advent of metagenomic sequencing the role of the gut microbiome in regulating human physiology, digestion, detoxification, and nervous and immune system development has been an increasing area of interest. Humans are now considered to have two separate genomes, one which is inherited, and the other (the microbial), which is acquired. The microbial genome has been noted to have far more genes (33 million) than the host genome (27 thousand). While the inherited genome is stable during the course of life, the microbial genome is dynamic and can be altered by a variety of factors, particularly during infancy. It is therefore important to understand the early life factors that influence the spatio-temporal acquisition of the microbiota for optimal health and disease prevention. This review provides an overview of the most pertinent factors relevant to pediatric health and development.

## 2. Factors That Shape Development of the Gut Microbiome in Infancy and Early Childhood

### 2.1. Parturition

Gut microbiome colonisation is highly variable in early infancy, and only a small subset of species that reach the GIT manage to colonise [15]. There is some controversy around when initial seeding of the gut microbiome occurs. However, evidence suggests initial colonisation of the GIT may begin as early as in utero [16] from species detected in the infant gut during the second trimester [17], with the mature, stable form of the gut microbiome being established at around three years of age [18]. Until recently, it was widely accepted that the foetus developed in a sterile environment and intestinal colonisation did not begin until birth (when the infant is first exposed to the mother’s vaginal and faecal microorganisms). However, recent studies have now identified microorganisms in the placenta [19], amniotic fluid [20], umbilical cord [21], and meconium [22]. Despite these observations, there is an ongoing debate that these published studies did not adequately control for contamination. However, the mounting evidence means it has now become generally accepted that seeding of the gut microbiome begins *in utero*, although the importance or effect of such early colonization is still unknown.

**Figure 1:**
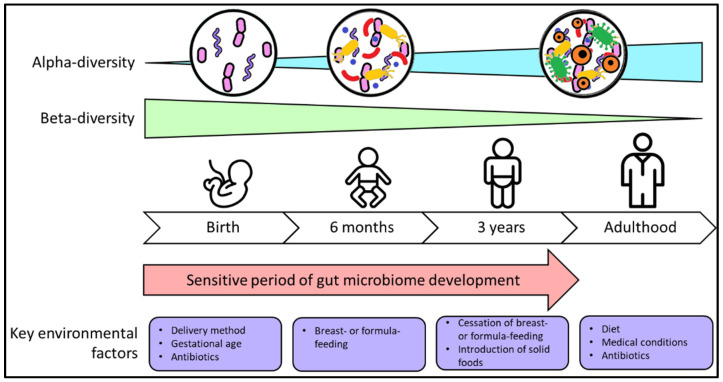
The developing gut microbiome and major influencing environmental factors. Alpha-diversity (diversity within one sample) increases as the gut microbiome develops. The beta-diversity (diversity between samples) decreases with age, indicating that gut microbiome differences are most variable between people during infancy, and become more similar n adulthood. The first three years of life represent a period of heightened plasticity where gut microbiome development is easily impacted by environmental factors.

During this time, an infant’s gut microbiome is highly sensitive and colonisation patterns are easily influenced by environmental exposures [23]. Thus, there is intense interest in ontogeny of the gut microbiome during this critical period of heightened plasticity in order to support optimal development and improved clinical outcomes into adulthood [24]. Initial colonisation of the infant gut is greatly affected by the mode of delivery (caesarean or vaginal delivery), with the next major transition points occurring postnatally around the commencement of breast-feeding, and the cessation of breast- or formula-feeding (Figure 1) [25].

Immediately after birth, the mother’s vaginal and feacal microbiomes are the major source of an infant’s gut microbiome colonisation. After parturition, the newborn gut microbiome undergoes rapid change. Skin-to-skin contact transfers microorganisms typically found on skin to the newborn [26], but the major determinant occurs with the introduction of breastmilk. Colostrum, the first milk received by the newborn, is very rich in antigen specific and non-antigen specific antimicrobials such as secretory IgA and lactoferrin, and is therefore expected to play a key role in the initial seeding of gut microbiota. Breastmilk is also an abundant source of bacteria as well as specific food for bacteria (Human Milk Oligosaccharides, HMOs) [27]. Within the first few months of life, with a large majority of infants on a milk-exclusive diet, the gut microbiome is primarily dominated by Bifidobacteria, which are highly adapted to utilise HMOs [28]. The cessation of a milk-based diet, and the introduction of solid foods drive the gut microbiome to a more ‘adult-like’ composition [25,29]; an adult gut microbiome is considered highly stable with minor fluctuations, except in cases of medical conditions, antibiotic use, or major diet changes [30,31].

### 2.2. Early Postnatal Life

A longitudinal study by Yassour et al., performed on 39 Finnish children aged 2 to 36 months of age collected monthly stool samples to provide insights into the development of the early infant gut microbiome [32]. They found that an overwhelming majority of infants in their study had significant levels (average abundances) of Enterobacteriaceae (25%), Bifidobacteriaceae (15%), and Clostridiaceae (8%) families, which decreased to a relative average abundance of 1%, 3%, and 2.5% by 18 months of age [32]. Once solid food is introduced, the gut microbiome shifts to a majority abundance of species from the *Bacteroides* genus, which is essential for starch digestion for a more complex diet [33]. Large population studies have mostly focused on gut microbiome research in children under 3 years of age, and adults aged 18 to 65 years of age. Thus, there is limited knowledge spanning the kindergarten and pre-school ages [34]. One study by Zhong et al. on 281 early school-aged children found that children dominated by Bifidobacterium had lower overall diversities and bacteria gene counts compared to children dominated by Bacteroides, or Prevotella [35]. The high levels of Bifidobacterium in school-age children’s gut microbiomes resembles that of an adult. Interestingly, they also found that the *Bifidobacterium* dominated group had an overall shorter breast-feeding duration [35]. A systemic review by Deering et al. has been conducted to address the gap in the literature of school-aged children’s gut microbiomes. They found the preadolescent gut is dominated by Firmicutes and Bacteroidetes [36].

### 2.3. Genetics

Recent studies have found that host genetics can influence the composition of the human gut microbiome which, in turn, can impact host metabolism. Development of the neonatal gut microbiome after birth is associated with interactions between the microbiota and the host immune system. Several studies, including genome-wide association studies, have found genome-wide significant associations for overall microbial variation [37,38,39]. A recent study by Xu et al. analysed single-nucleotide polymorphism (SNP) heritability of 1475 Chinese participants. They found that *Desulfovibrionaceae* and *Odoribacter* had significant heritability estimates of 0.456, and 0.476, respectively [40], which is consistent with previous research [41]. Genetic variation between individuals can drive variation in microbiome composition, and twin studies allow us to understand to what extent this variation is genes or environment driven. One study by Goodrich et al. assessed the heritability of the gut microbiome using a twin study and noted that monozygotic twins have more similarities in gut microbiota than dizygotic twins with operational taxonomic unit (OTU) heritability estimates ranging from 0.2 to 0.4 [42]. OTUs are clusters of similar sequence variants used to classify closely related species. They also found that there are many microbial taxa whose abundances were influenced by host genetics, the most heritable belonging to the *Christensenellaceae* family [42].

Since large-scale studies have been conducted, the gut microbiome has been revealed to be highly personalised, even among healthy individuals [43]. It is well-known that genetic variation exists between human populations and across geographic locations. Gut bacterial communities have also been shown to vary across human populations [15]. A recent study by Deschasaux et al. used 16S sequencing to analyse stool samples from over two thousand adults from the six largest ethnic groups in Amsterdam. They concluded that ethnic differences from ethnic groups residing in the same geographic location accounted for a larger proportion of the variation of gut microbiome than other major influencing factors, including diet [44]. These findings suggest that host-intrinsic genetic diversity influences gastrointestinal colonisation patterns, as well as environmental factors and dietary patterns. Deschasaux et al. noted that despite differences in gut microbiome composition between ethnic groups, there were 21 microbial taxa that were ubiquitous among all 2084 participants across the different ethnic groups [44], suggesting individuals from the same ethnic group tend to have more similarities in gut microbiome composition than individuals from different ethnicities [45]. This supports the notion that despite high levels of inter-individual variation in gut microbiome profiles, there are some ubiquitous species amongst populations that are important for human health and therefore common across different population. Understanding the factors that impact gut microbiome development and composition, including genetics, is critical to recognize the role of the gut microbiome in disease progression.

### 2.4. Infant Feeding

Infant feeding of breast or formula milk has significant influence on the composition of the early gut microbiota in the first year of life [46]. Extensive research has shown the short-term and long-term benefits of breast-feeding. Breast milk contains IgA and IgG immunoglobulins, antimicrobial compounds, lymphocytes, growth factors, vitamins, and cytokines, which are transferred to the infant and expected to aid the development of a healthy immune system and to influence shaping of the gut microbiome [47,48]. Breast-fed infants tend to have a gut microbiome enriched with Lactobacillus, *Staphylococcus*, and *Bifidobacterium*, compared with formula-fed infants with microbiomes enriched with *Roseburia*, *Clostridium*, and *Anaerostipes* [25]. Breast-fed infants tend to have less diverse gut microbiomes but higher levels of *Bifidobacterium* species, including *Bifidobacterium breve*, *Bifidobacterum bifidum*, and *Bifidobacterium longum*, which thrive on human milk oligosaccharides (HMOs) and are highly specialised at digesting HMO’s in breast-milk [49]. HMOs vary from mother to mother based on genetic factors. The majority of mothers secrete oligosaccharides into their breast milk which is based on the presence of the FUT2 gene, located on chromosome 19 [50]. Some mothers have a benign mutation of this gene, making them non-secretors, and reducing the diversity of *Bifidobacterium* in their breast-fed children [51]. However, one large cohort study by Turpin et al. found no association between FUT2 genotype and the resulting phenotype is not associated with gut microbiome composition [52].

### 2.5. Introduction of Solid Foods

The next major transition point in the infant gut microbiome occurs during the weaning period, when solid foods are introduced. Solid food introduction drives a shift in the microbiome composition away from being dominated by *Bifidobacterium* to being dominated by *Bacteroides* and *Firmicutes* species [53]. This change in species abundance allows for a change away from bacterial genes used for lactate digestion, to genes better suited to carbohydrate digestion [18]. These alterations continue until three years of age and mediate the transition toward an adult-like gut microbiome with respect to composition and diversity. From this point, the gut microbiome remains relatively stable with the exception of long-term dietary changes, dysbiosis caused by disease, or antibiotic exposure. Interestingly, the introduction of solid foods too early (before three months of age) has been associated with changes in the infant gut microbiome composition by increasing diversity and butyrate concentrations, and has been linked to an increased risk of childhood obesity, immune disorders, and oxidative stress [54].

## 3. Environmental and Lifestyle Factors That Contribute to Gut Dysbiosis

### 3.1. Delivery Method

The particular species initially colonising the gut microbiome of newborn babies is greatly affected by mode of delivery (caesarean delivery or vaginal delivery), and is one of the most important determinants for newborn gut microbiome development [55]. Studies have found that caesarean deliveries can negatively impact the infant gut microbiome and subsequently immune system development by altering the type and reducing the diversity of initial colonising microorganisms. Immune and allergic disease risk are also higher in caesarean born infants, in particular asthma, arthritis, inflammatory bowel disease, and immune deficiencies [56]. This could be a result of caesarean deliveries contributing to gut dysbiosis. Some strategies have been investigated to repair the newborn gut microbiota following caesarean deliveries, such as vaginal seeding which have begun to be introduced in certain hospitals [57]. An infant is first introduced to a wide variety of microorganisms during delivery, where the infant is exposed to a few specific types of bacteria that colonise the mother’s birth canal. In some cases, a caesarean delivery may be necessary, and the infant is exposed to different set of microorganisms.

The World Health Organisation (WHO) recommends that caesarean deliveries remain at a rate under 15%. However, in developed countries, the rate of caesarean deliveries has been increasing. Data from 150 countries has shown increasing rates of caesarean deliveries from 6.7% to 19.1% from 1990 to 2014 [58]. Vaginal birth is associated with a gut microbiome signature in newborns, as measured from early stool samples, that is similar to the maternal vaginal microbiota, and caesarean births are associated with species that more closely resemble skin taxa [59]. In the first week after birth, caesarean born babies tend to have decreased levels of *Bacteroides*, *Lactobacillus*, and *Bifidobacterium*, and increased amounts microorganisms associated with skin microbiomes, such as *Staphylococcus*, *Streptococcus*, and *Propionibacterium* [60]. After the first week of life, caesarean born infants have reduced *Bifidobacterium*, and higher levels of *Klebsiella*, *Haemophilus*, and *Veillonella* [61]. After the first month of life, differences between the delivery methods no longer appear significant, but with some differences in *Lactobacillus*, *Bacteroides*, and *Bifidobacterium* still being detectable [62]. By 6 months of age, the colonisation patterns are near identical between vaginal and caesarean born babies. However, *Bacteroides* and *Parabacteroides* remain higher in vaginally born infants, and *Clostridium* species’ are more abundant in caesarean born infants [55].

Caesarean births often mean infants have delayed contact with their mothers, a delayed start to breastfeeding, and the sterile environment of the operating room can promote colonisation of hospital flora, which has been associated with a higher risk of developing respiratory disorders [63]. In many hospitals, it is also routine to administer (via a drip) antibiotics to the mother during delivery, leading to early infant exposure to antibiotics during birth. One pilot study by Imoto et al. in 2018 found that maternal antimicrobial use during delivery had a stronger impact on the newborn gut microbiome than delivery mode, specifically reduced colonisation of Bifidobacteria [64].

Interestingly, there is a difference between elective caesareans and emergency caesareans in terms of infant gut microbiome signatures. In fact, the gut microbiome signatures between caesareans that were preceded by the beginning of labour are more closely related to that of vaginal births than elective caesareans. In an elective surgery, infants are not exposed to the intrauterine inflammatory cytokines promoted by the immune response of labour. These cytokines have been linked to stimulating the infant’s immune system development [65].

### 3.2. Antibiotic Use

Antibiotics are one of the most prescribed medications and have numerous benefits. However, their use has also been linked to both short-term and long-term negative impacts on health, such as an increased risk of autoimmune diseases and asthma [66,67]. Infants, and particularly preterm infants, are more susceptible to bacterial infections and sepsis than adults. Antibiotic administration in early life results in decreased diversity and changes to the abundance of particular microbial species in the gut, specifically *Bifidobacterium*, decreased resistance to opportunistic pathogens and increased antibiotic resistance [68]. The disruption of the natural assembly and reduced diversity of the gut bacteria caused by antibiotics could potentially be linked to the adverse health outcomes. *Clostridium difficile* is one of the most common infections across all age groups following antibiotic use, representing 15–25% of antibiotic associated diarrhoea and the majority of antibiotic associated-colitis cases [69]. A systematic review conducted by Zimmermann and Curtis investigated the effect of systemic antibiotics on the intestinal microbiome in humans. They found that antibiotics have a profound and often persisting effect on the gut microbiome [70]. One study performed in adults by Hagan et al. found that alterations in the gut microbiome driven by antibiotics adversely affected the responsiveness to the influenza vaccination in humans [71]. They found that antibiotic use significantly altered the transcriptional and metabolic processes of peripheral blood mononuclear cells (PBMCs) [71]. It is still unclear on whether the effects of antibiotics on the gut microbiome persist long-term, and what the extent of the effects may be. However, the specific effects on the gut microbiome depends on the antibiotic class, route of administration, dose, duration, and spectrum of activity (broad or narrow) [70]. When the gut microbiome is exposed to antibiotics, the microorganisms respond by harbouring and exchanging antibiotic resistance genes (as the resistome). A paper by Palleja et al. used shotgun metagenomics to analyse the reduction and regrowth of the adult gut microbiome over six months, after a course of last-resort antibiotics [72]. They found that immediately following antibiotics, there was an initial increase in Enterobacteria and other pathogenic species such as *Enterococcus faecalis*, and *Fusobacterium nucleatum*, and a reduction in *Bifidobacterium* species and butyrate producers [72]. Within one and a half months of the intervention, the gut microbiomes of the participants recovered to almost baseline. However, there were nine species present in all participants before the treatment that were no longer present after six months [72]. This suggests that the gut microbiome in healthy adults is resilient to the long-term effects of antibiotics, but there may be some lasting effects, particularly in long-term antibiotic exposure. A recent longitudinal study by Korpela et al. analysed stool samples from infants following a course of antibiotics [73]. They found that following a single course of antibiotics, infant gut microbiomes had varied abundances of *Bifidobacteria*, *Enterobacteria*, and *Clostridia*, which persisted for several months, with *Bifidobacteria* levels having the most significant variation [73]. A recent study by Sun et al. found that mice treated with antibiotics had up-regulated gene expression of various cytokines in the colon [74]. This suggests that antibiotic-induced changes in the gut might contribute to inflammation responses.

Gaining an understanding on the role antibiotics play in affecting the composition of the gut microbiome will help to minimise the damage caused by antibiotic treatment by tailoring antibiotic treatment and probiotic use, which is already underway in adults. Currently, sequencing data can determine taxa within the gut microbiome but more advanced sequencing techniques are required to provide analysis on antibiotic resistance genes in the gut microbiome to better inform antibiotic therapies, particularly in early life, when the gut microbiome is more susceptible to the effects of antibiotics.

### 3.3. Other Factors

There have been several observational studies demonstrating that exposure to pets or siblings early in life can protect against allergic disease [75,76,77]. This is often coupled with the “hygiene hypothesis”, which postulates that limiting exposure to varied microorganisms can be sub-optimal for immune development in early life. Contact with household pets and siblings is thought to promote a more diverse gut microbiome in young children, which is known to protect against atopy [76]. One study by Tun et al. found that infants exposed to pets both prenatally and postnatally had a higher abundance of *Ruminococcus*, and *Oscillopria* (studied in both caesarean and vaginal deliveries), which are negatively associated with childhood obesity and atopy [78]. Thus, the body of evidence based upon the effects of household furry pets on the infant gut microbiome is contentious. However, these studies support the notion that the infant gut microbiome is highly plastic.

In recent decades, mortality rates of very preterm infants have been substantially reduced. However, these reduced mortality rates do not correlate with reduced morbidities. Preterm infants have an altered gut microbiome and, when paired with their immature immune response, trigger pro-inflammatory and counter-inflammatory responses [79]. Preterm infants tend to have delayed *Bifidobacterium* colonisation, and a higher abundance of *Staphylococus*, and *Enterobacteriaceae* [80]. Preterm neonates also have a tendency toward pro-inflammatory responses, which has been associated with higher levels of *Enterobacter*, *Enterococcus*, and *Lactobacillus* [81]. One study found that extremely preterm neonates (born before 28 weeks of age) had higher levels of *Lactobacillus* in the infant’s meconium, which dominates the maternal vaginal microbiome than very preterm neonates (born after 28 weeks of age); this was independent of mode of delivery [82]. This supports the notion that the initial seeding of the GI tract is influenced by the maternal microbiome. Preterm infants are also more likely to be hospitalised in intensive care units, and have different nutritional requirements, which exposes the infant to the sterile hospital environment and, in some cases, antibiotics [83,84]. Preterm infants who were born at a very-low-birth-weight (VLBW) are more likely to develop intestinal microbial dysbiosis with a low gut microbiome diversity, reduced levels of beneficial microorganisms, and increased levels of opportunistic pathogens [85]. This has been theorised to be caused by the interrupted intrauterine development and may be linked to increased inflammation of the gut. VLBW infants are also often separated from their mothers and placed in incubators to aid in body temperature regulation.

## 4. Links between Gut Dysbiosis and Disease

### 4.1. GIT-Related Disorders

If there are disruptions in the microbial composition of the gut, the homeostatic balance can be interrupted. This could lead to an imbalance between beneficial and potentially pathogenic bacteria (i.e., dysbiosis). In general, dysbiosis is characterized into different types such as a loss of beneficial microorganisms, an over production of pathogenic microorganisms, or a loss of overall diversity in the gut. These different types of gut dysbiosis may occur individually or all at the same time. Gut dysbiosis has been attributed to a wide range of diseases such as IBD, obesity, allergic disorders, Type 1 diabetes, and autism [86]. The most prevalent forms of IBD are Crohn’s disease (CD) and ulcerative colitis (UC), which are both characterized by chronic relapsing inflammation of the intestinal mucosa, and different gut microbiome signatures [87]. Although the specific causes of CD and UC are largely unknown, increasing evidence has linked gut dysbiosis to IBD [88]. Patients presenting with IBD typically have overall decreased gut microbiome diversity and stability, as well as decreased abundance of specific *Firmicutes*, and an increase in *Bacteroidetes*, and *Enterobacteriaceae* [89]. There is still an unresolved argument if intestinal dysbiosis is a direct cause for inflammation in IBD, or a result of the disrupted GIT. As well as IBD, the gut microbiome has been linked to obesity, type 2 diabetes, colorectal cancer, and irritable bowel syndrome (IBS). In IBS, colorectal cancer, and coeliac disease, changes in gut microbiome composition have been described when compared to healthy controls [90]. However, there have not been any consistent patterns of microbial change observed in the literature.

### 4.2. Central Nervous System-Related Disorders

**Figure 2:**
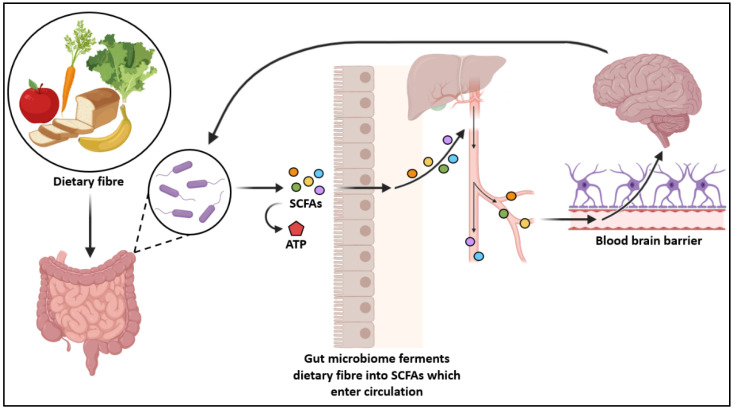
The gut-brain-axis showing the bidirectional relationship between the gut microbiome and the nervous system. The gut microorganisms ferment dietary fibre which produces SCFAs. The SCFAs are proposed to enter the circulatory system and into the brain via the blood brain barrier.

Gut dysbiosis has also been linked to extra-intestinal diseases, especially those that are affected by the gut-brain-axis, impacting the central nervous system (CNS). The gut-brain-axis is the bidirectional communication between the nervous system and the gut microbiome. Recent research has found that the dysbiosis of the gut microbiome can alter the development and regulation of the hypolalamic-pituitary-adrenal (HPA) axis, which functions as a physiological adaptive response to psychological stressors [91]. In infants, colonisation is important for the development of the enteric nervous system and the development and function of the CNS [92]. A study by Bravo et al. found that long-term treatment with probiotics decreased levels of stress-induced corticosteroids, symptoms of depression and anxiety [93]. Gut microbiota also play an important role in fermenting carbohydrates and proteins to produce metabolites such as SCFAs, which regulate microglial homeostasis [94]. Primarily consisting of acetate, propionate, and butyrate, SCFAs can enter systemic circulation, as well as other tissues and organs [95]. They may then be able to cross the blood-brain-barrier into the central nervous system (Figure 2). Evidence supporting this notion is lacking in human studies. However, animal studies have provided evidence on the effect SCFAs have in CNS disorders and psychological functioning [96]. Animals with no microbial colonisation (germ-free), or with a removed microbiome (antibiotic-treated) have different neurophysiology and behavior when compared to mice with typical colonisation patterns. Only some of these phenotypes can be restored postnatally. This suggests a role of the maternal gestational microbiome in the development of nervous system of their offspring [97]. Recent research has begun investigating feacal microbiota transplantation as a strategy to alleviate gut microbiome changes associated with disorders such as Alzheimer’s disease [98].

The exact causes of autism spectrum disorder (ASD) are still largely unknown. However, the gut microbiome has been proposed to play a role in autism development. Children diagnosed with ASD have been found to have dysbiotic gut microbiomes and altered SCFA levels [99,100,101,102]. One study found that mice treated with *Lactobascillus rhamnosus* showed reduced anxiety and depression symptoms, which was not observed in mice with a vagus nerve ablation, suggesting a role of the vagus nerve in the gut-brain-axis [93]

### 4.3. Immunity

In early life, gut microbiome colonisation is also associated with immune system development. Studies involving germ-free mice have shown loss of immune function in the absence of a normal microbiome [103]. In infants, immune factors such as the maternal antibodies IgG and IgA from the placenta and breast-milk play an important role in the selection of intestinal bacteria [47]. The altered development of the immune system has been linked to the onset of several autoimmune and allergic diseases, as revealed by cohort studies, some of which has found that infants with atopic dermatitis have low overall bacterial diversity, and a low abundance of *Bifidobacterium* and *Bacteroides*, as well as an increased abundance of *Enterobacteiaceae* [104]. Fujimura et al. also reported that reduced abundance of Bifidobacterium coincided with infants that were at higher risk of atopic dermatitis and asthma, as well as high abundances of some fungi such as *Candida* and *Rhodotorula*, and high levels of pro-inflammatory metabolites [105]. Food allergies are characterized by inflammation of the skin or GIT in response to particular foods and are thought to impair oral tolerance. Ling et al. found that infants who develop food allergies had distinct gut microbiome signatures at one year of age, with low abundances of *Bacteroides* [106]. Contradictory results were found by Azad et al. who investigated infants with food sensitivities compared to infants without, and found they had low diversities and high abundances of *Enterobacteriaceae* at three months of age, and high abundances of *Bacteroides* at one year of age [107]. Infants who have early dysbiotic colonisation of *Escherichia coli* are also at an increased risk of developing eczema [108], and *Clostridium difficile* colonisation in the first month of life is highly correlated with asthma at six to seven years of age [109].

## 5. Conclusions

There is a need for future research and a better understanding on the role gut dysbiosis plays in inflammatory disease to inform potential mitigation strategies. This research could lead to insights on ideal gut microbiome signatures, and will inform interventions (for example, supplementation with pre- and pro-biotics, dietary interventions, or feacal microbiota transplantations in more severe gut dysbiosis cases).

Numerous environmental factors culminate to influence overall patterns of microbial colonisation of the GIT in early life. Environmental influences including delivery mode, infant feeding patterns and methods, dietary patterns, antibiotic use, and exposure to pets and siblings may influence overall composition and diversity. Studies continue to investigate other factors in the built environment such as tap water quality, exposure to green space, and their role in shaping gut microbiome development. With the availability of next-generation sequencing technology, it is anticipated that many new discoveries will shed new light on the ontogeny of the gut microbiome and its role in health and disease.
